# A combination of indol-3-carbinol and genistein synergistically induces apoptosis in human colon cancer HT-29 cells by inhibiting Akt phosphorylation and progression of autophagy

**DOI:** 10.1186/1476-4598-8-100

**Published:** 2009-11-12

**Authors:** Yoshitaka Nakamura, Shingo Yogosawa, Yasuyuki Izutani, Hirotsuna Watanabe, Eigo Otsuji, Tosiyuki Sakai

**Affiliations:** 1 Department of Molecular-Targeting Cancer Prevention, Graduate School of Medical Science, Kyoto Prefectural University of Medicine, Kawaramachi-Hirokoji, Kamigyo-ku, Kyoto 602-8566, Japan; 2 Division of Digestive Surgery, Department of Surgery, Kyoto Prefectural University of Medicine, Kawaramachi-Hirokoji, Kamigyo-ku, Kyoto 602-8566, Japan

## Abstract

**Background:**

The chemopreventive effects of dietary phytochemicals on malignant tumors have been studied extensively because of a relative lack of toxicity. To achieve desirable effects, however, treatment with a single agent mostly requires high doses. Therefore, studies on effective combinations of phytochemicals at relatively low concentrations might contribute to chemopreventive strategies.

**Results:**

Here we found for the first time that co-treatment with I3C and genistein, derived from cruciferous vegetables and soy, respectively, synergistically suppressed the viability of human colon cancer HT-29 cells at concentrations at which each agent alone was ineffective. The suppression of cell viability was due to the induction of a caspase-dependent apoptosis. Moreover, the combination effectively inhibited phosphorylation of Akt followed by dephosphorylation of caspase-9 or down-regulation of XIAP and survivin, which contribute to the induction of apoptosis. In addition, the co-treatment also enhanced the induction of autophagy mediated by the dephosphorylation of mTOR, one of the downstream targets of Akt, whereas the maturation of autophagosomes was inhibited. These results give rise to the possibility that co-treatment with I3C and genistein induces apoptosis through the simultaneous inhibition of Akt activity and progression of the autophagic process. This possibility was examined using inhibitors of Akt combined with inhibitors of autophagy. The combination effectively induced apoptosis, whereas the Akt inhibitor alone did not.

**Conclusion:**

Although *in vivo *study is further required to evaluate physiological efficacies and toxicity of the combination treatment, our findings might provide a new insight into the development of novel combination therapies/chemoprevention against malignant tumors using dietary phytochemicals.

## Background

Malignant tumors are a leading cause of death in many countries and chemoprevention has become an important issue. Since chemoprevention with nontoxic agents could be one approach to reducing the incidence of cancers, anticancer effects of dietary phytochemicals including polyphenols have recently been studied [1,2]. It has been suggested that a combination of agents is more effective than any single constituent in achieving chemopreventive effects [3]. For this reason, studies on synergistic effects of different phytochemicals might contribute to the chemopreventive strategies against malignant tumors.

Genistein is a soy-derived isoflavone with multiple biochemical effects, including the alteration of cell cycle-regulatory kinase activities [4,5]. Previous studies indicated that genistein induced apoptosis, enhanced the induction of apoptosis by chemotherapeutic agents, and increased radiosensitivity in several cancer cell lines [4,6]. Genistein is also known as an estrogen receptor (ER) agonist, and that genistein can antagonise the proliferation of breast cancer cells by estradiol [7]. However, most transcription activation bioassays are not able to show an estrogen receptor antagonism of genistein, and genistein acts additive to estradiol in theses systems [8]. It is therefore controversial whether anti-proliferative effect of genistein is ER-dependent or not [9].

Indole-3-carbinol (I3C), derived from Cruciferous vegetables, has been shown to suppress the growth of various tumor cells including colon cancer cells by arresting the cell cycle at G1/S and inducing apoptosis *in vitro *[10], targeting molecules such as Bcl-2, mitogen-activated protein kinase (MAPK), cyclin D1, and the cyclin-dependent kinase (CDK) inhibitors p21, p27 [10] and p15 [11]. I3C is also known as an androgen receptor (AR) antagonist. Previous reports suggest that I3C is able to inhibit AR mediated proliferation of prostate cancer cells [12].

Both genistein and I3C have been reported to down-regulate the phosphatidylinositol 3-kinase (PI3K)/Akt signaling pathway. Akt is a serine/threonine protein kinase, also known as protein kinase B (PKB), which plays a critical role in suppressing apoptosis [13,14] by regulating its downstream pathways [15-18]. On the other hand, Akt also phosphorylates mammalian target of rapamycin (mTOR), which has been reported to inhibit the induction of macroautophagy (hereafter referred to as autophagy) [19,20].

Autophagy is the regulated process by which cytoplasmic constituents are recruited to lysosomes for degradation [19,21,22]. The autophagic pathway begins with the formation of a double-membrane vesicle called the "autophagosome" which engulfs organelles or long-lived proteins and matures into an acidic single-membrane autophagosome that fuses with a lysosome to become the "autolysosome", whose content is degraded [20,21]. Recently, the relationship between autophagy and apoptosis has been studied extensively [23-26]. Although the molecular mechanism underlying this interconnection is still obscure, several reports have suggested autophagy to be induced by anticancer treatments with γ-irradiation or chemotherapeutic agents, to protect cancer cells from apoptosis [20,26-28]. Thus, inhibition of autophagy may induce apoptosis [29-36].

We here found for the first time that co-treatment with I3C and genistein synergistically induced apoptosis in human colon cancer HT-29 cells by simultaneously inhibiting the phosphorylation of Akt and progression of the autophagic process.

## Results

### Co-treatment with I3C and genistein synergistically inhibits the viability of HT-29 cells

**Figure 1:**
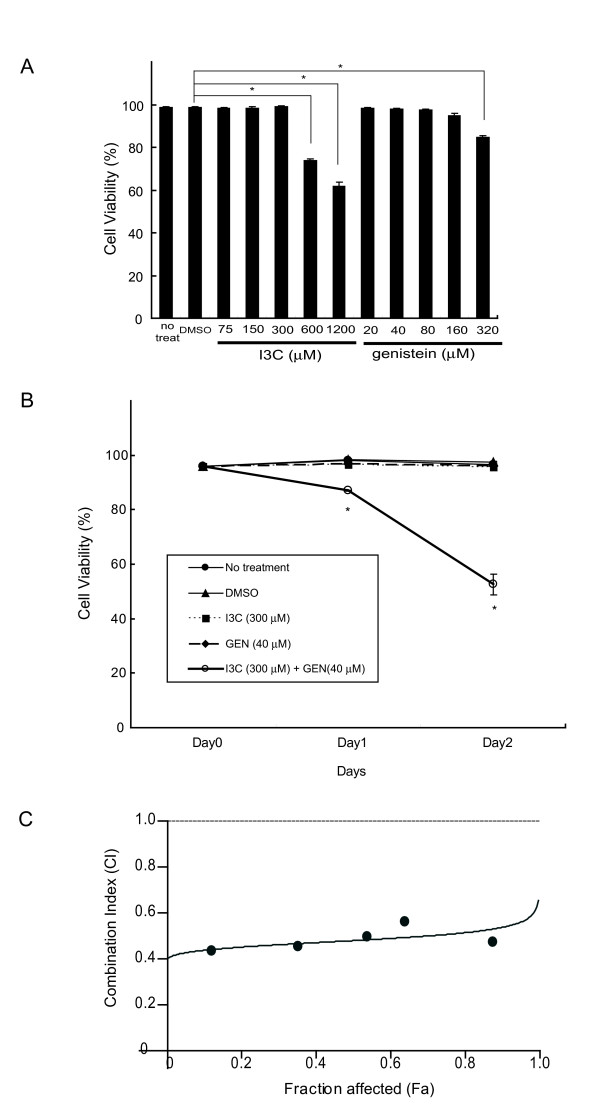
**Inhibitory effects of I3C and genistein on cell viability in human colon cancer HT-29 cells**. **A**, Viability of HT-29 cells 48 h after exposure to increasing doses of I3C or genistein as indicated. **B**, Time-dependent viability of HT-29 cells not treated (black circle) or treated with DMSO (black triangle), I3C (300 μmol/L) (black square), genistein (40 μmol/L) (black triangle), or a combination of I3C (300 μmol/L) and genistein (40 μmol/L) (white circle) for up to 2 days. The cell viability assay was performed as described in Materials and Methods. *Columns *and *points*, mean (n = 3); *bars*, SD. *, *P *< 0.05, significantly different compared with the DMSO-treated control. *GEN*, genistein. **C**, HT-29 cells were exposed to increasing concentrations of I3C and genistein, either alone or in a fixed ratio of 7.5:1, for 48 h, as described in Materials and Methods. The Fa-CI plot was constructed using experimental data points (dark circles) and by simulating CI values over the entire range of Fa values (line) using CalcuSyn software. Synergy, additivity, and antagonism are defined as CI<1, CI = 1, and CI>1, respectively.

To examine the effect of I3C or genistein on the human colon cancer cell line HT-29, a cell viability assay was first performed. HT-29 cells were treated with I3C at concentrations ranging from 75 μmol/L to 1200 μmol/L or with genistein at 20 μmol/L to 320 μmol/L, for 48 h. As shown in Fig. 1A, neither I3C at up to 300 μmol/L nor genistein at up to 160 μmol/L had any significant inhibitory effect on cell viability. The time dependent-suppressive effect of I3C and/or genistein on viability was assessed further and remarkable suppression was observed when 300 μmol/L of I3C and 40 μmol/L of genistein were combined, reducing the cell viability to 87.0% after 24 h and 52.6% after 48 h, whereas each agent alone had no inhibitory effect on cell viability over the 48 h (Fig. 1B). To further determine whether the inhibitory effects were synergistic, we analyzed CI value of the combination using CalcuSyn software. As shown in Fig. 1C, the CI values remained < 1 over the entire range of Fa values, indicating that I3C and genistein are synergistic in terms of inhibitory effect on cell viability.

### Co-treatment with I3C and genistein synergistically induces apoptosis

**Figure 2:**
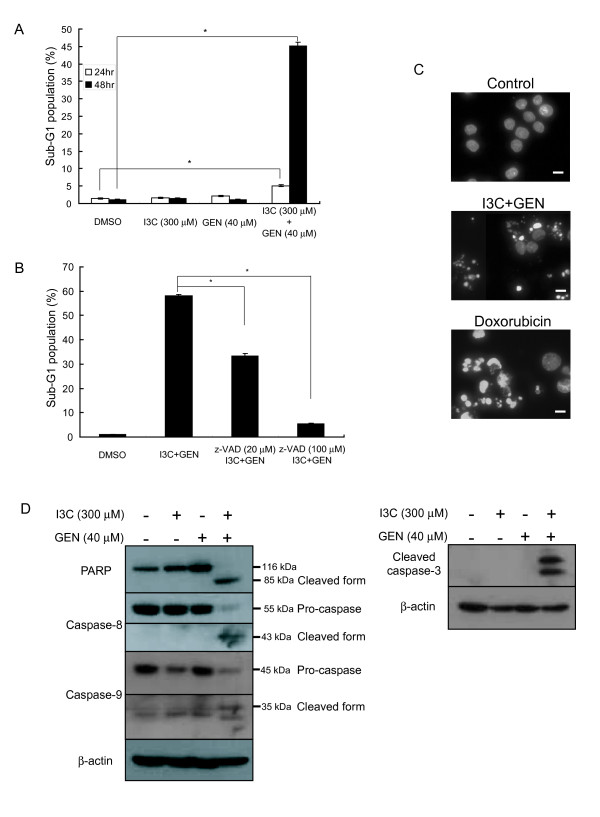
**Effects of co-treatment with I3C and genistein on apoptosis in HT-29 cells**. **A**, The sub-G1 population was assessed by flow cytometry as described in Materials and Methods after exposure to the indicated agents for 24 or 48 h. *Columns*, mean (n = 3); *bars*, SD. *, *P *< 0.05, significantly different compared with the DMSO-treated control. **B**, HT-29 cells were exposed to DMSO or a combination of I3C (300 μmol/L) and genistein (40 μmol/L) for 48 h or pretreated with z-VAD-fmk (20 or 100 μmol/L) for 2 h followed by exposure to the combination for 48 h. The sub-G1 population was quantified by flow cytometry as described in Materials and Methods. *Columns*, mean (n = 3); *bars*, SD. *, *P *< 0.05, significantly different compared with the combined treatment. **C**, Cells were exposed to DMSO (control), a combination of I3C (300 μmol/L) and genistein (40 μmol/L), or doxorubicin (1 μmol/L) for 48 h, stained with DAPI, and observed under a fluorescence microscope as described in Materials and Methods. Doxorubicin was used as a positive control to induce apoptosis. *Bars*, 20 μm. **D**, HT-29 cells were exposed to the indicated agents for 48 h. The expression of PARP, caspase-8, caspase-9 and cleaved caspase-3 proteins was analyzed by western blotting. β-actin was used as a loading control. -, treated with DMSO. *GEN*, genistein.

We next investigated whether the cell death induced by the co-treatment with I3C and genistein could be apoptosis. We analyzed the cell cycle distribution by flow cytometry and observed a significant increase in the sub-G1 population among the cells co-treated with I3C and genistein for 48 h. In contrast, neither I3C nor genistein alone had any effect on the sub-G1 population (Fig. 2A). Moreover, the increase in the sub-G1 population caused by the co-treatment was abrogated by the pan-caspase inhibitor z-VAD-fmk (Fig. 2B), suggesting it to be due to caspase-dependent apoptosis. To further characterize this cell death, we performed DAPI staining. As shown in Fig. 2C, cells co-treated with I3C and genistein showed nuclear fragmentation and chromatin condensation. Collectively, these features are characteristic of apoptosis. To confirm these results at the molecular level, we investigated the cleavage of poly (ADP-ribose) polymerase (PARP) or activation of caspase-3, caspase-8, and caspase-9 by western blotting. As shown in Fig. 2D, neither I3C nor genistein alone could activate these caspases, although in combination they clearly cleaved PARP and these caspases to their active forms. Collectively, these results provide evidence that the cell death induced by the co-treatment with I3C and genistein is caused by caspase-dependent apoptosis.

### Co-treatment with I3C and genistein reduces phosphorylated Akt and its downstream targets

**Figure 3:**
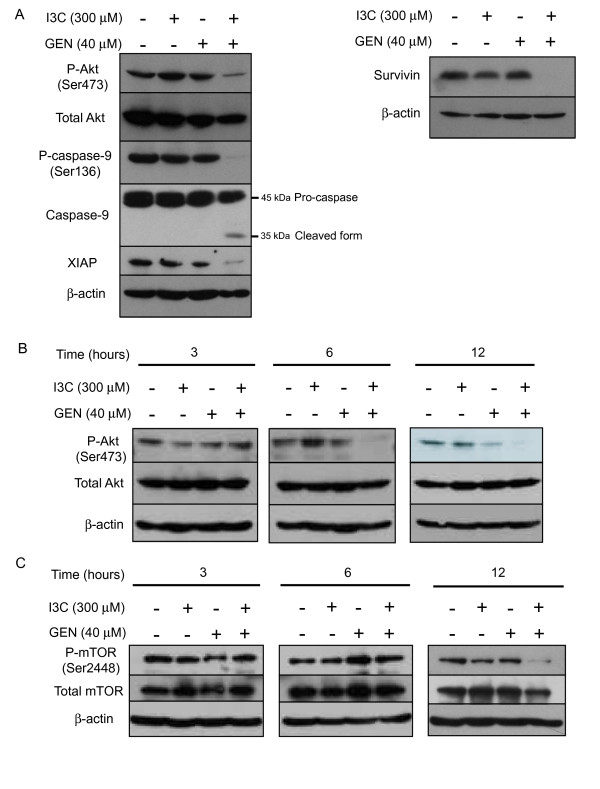
**Expression of Akt and its downstream effectors following co-treatment**. **A**, After 48 h of exposure to the indicated agents, cell lysates were subjected to western blotting using antibodies against phospho-Akt (Ser473), total Akt, phospho-caspase-9 (Ser136), caspase-9, XIAP or survivin. **B **and **C**, After exposure to the agents for the periods indicated, cell lysates were subjected to western blotting with anti-phospho-Akt (Ser473), anti-Akt (**B**), anti-phospho-mTOR (Ser2448), and anti-mTOR (**C**) antibodies. β-actin was used as a loading control. -, treated with DMSO. *GEN*, genistein.

Previous reports indicated that either I3C or genistein inhibited Akt activity through a reduction in its phosphorylation [4,10]. Once activated, Akt transduces signals to downstream targets that control cell survival and inhibit apoptosis [13,14]. To assess the involvement of the Akt pathway in the apoptosis induced by the co-treatment with I3C and genistein, the level of phosphorylated Akt protein was investigated by western blotting. As shown in Fig. 3A and 3B, phosphorylated Akt started to decrease 6 h after the co-treatment. Twelve hours after the co-treatment caspase-3 started to be activated [see Additional file 1], suggesting that dephosphorylation of Akt occurs before apoptosis.

In addition, we further investigated the expression of phosphorylated caspase-9, a downstream target of Akt, and found that the co-treatment significantly reduced the level of phospho-caspase-9 (Ser196), resulting in activation of caspase-9. Since X chromosome-linked inhibitor of apoptosis protein (XIAP) and survivin, inhibitor of apoptosis protein (IAP) family members, have been recently reported to be activated by Akt [17,18], we further investigated the expression of the proteins. As shown in Fig. 3A, both XIAP and survivin expression was markedly downregulated by the combined treatment, consistent with the inhibition of Akt phosphorylation by the treatment.

Since mTOR is another downstream effector of Akt, we further investigated phosphorylated mTOR expression by western blotting. As shown in Fig. 3C, the co-treatment clearly reduced the phosphorylated mTOR at 12 h.

### Co-treatment with I3C and genistein induces autophagosome formation

**Figure 4:**
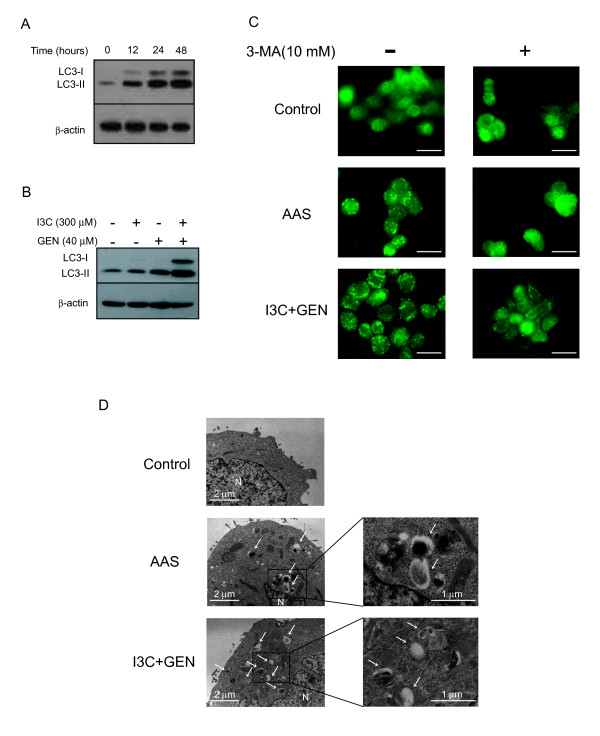
**Detection of autophagosomes following co-treatment**. **A**, After exposure to a combination of I3C (300 μmol/L) and genistein (40 μmol/L) for the periods indicated, cell lysates were subjected to western blotting with an anti-LC3 antibody. β-actin was used as a loading control. **B**, After exposure to the indicated agents for 48 h, cell lysates were subjected to western blotting with an anti-LC3 antibody. β-actin was used as a loading control. -, treated with DMSO: **C**, HT-29 cells were exposed to DMSO (control) or a combination of I3C (300 μmol/L) and genistein (40 μmol/L) for 12 h with or without 10 mmol/L of 3-methyladenine (3-MA). As a positive control for the induction of autophagy, cells were subjected to amino acid starvation (AAS) by incubating them in amino acid-deprived medium for 12 h. After the incubation, cells were subjected to immunofluorescent staining for LC3 as described in Materials and Methods. *Bars*, 20 μm. **D**, HT-29 cells were exposed to DMSO (control) or a combination of I3C (300 μmol/L) and genistein (40 μmol/L) in complete medium or subjected to AAS for 12 h. The cells were then harvested and subjected to transmission electron microscopy as described in Materials and Methods. *White arrows*, autophagic vesicle. *N*, nucleus. *GEN*, genistein.

Several reports indicate that PI3k/Akt signaling negatively regulates autophagy through mTOR [19,37]. Recent studies have shown that the inhibition of Akt and its downstream target mTOR contributes to the initiation of autophagy [38,39]. To investigate whether co-treatment with I3C and genistein could promote autophagy via inhibition of the Akt/mTOR pathway, we measured the expression of microtubule-associated protein-1 light chain-3 (LC3) protein by western blotting. During autophagy, cytosolic LC3-I is conjugated with phosphatidylethanolamine and converted to LC3-II, and this process is essential for the formation of autophagosomes. Since LC3-II is present specifically on isolation membrane and autophagosomes, its amount correlates with the number of autophagosomes and serves as an indicator of their formation [40]. We found an enhancement of LC3-II expression in the cells co-treated with I3C and genistein from 12 h up to 48 h (Fig. 4A). Moreover, the up-regulation of LC3-II did not occur in the cells treated with either agent alone (Fig. 4B).

We next investigated the localization of endogenous LC3 by immunofluorescent staining. It has been suggested that LC3 is recruited to the autophagic membrane during the induction of autophagy, and the formation of autophagosomes is reflected by a punctate distribution of LC3 [41]. As shown in Fig. 4C, only a few LC3-positive puncta were observed in HT-29 cells treated with DMSO control. On the other hand, numerous LC3-positive puncta were observed in the cells subjected to amino acid starvation, by which autophagy has been induced [21]. In HT-29 cells co-treated with I3C and genistein, numerous LC3-positive puncta were observed, suggesting the accumulation of autophagosomes. This result is consistent with LC3-II protein levels detected by western blotting (Fig. 4A and 4B). Moreover, the numbers of puncta were reduced by 10 mmol/L of 3-MA (an autophagy inhibitor) [42], as expected. These results suggest that the numbers of puncta reflect autophagosomes, consistent with a previous report [41].

We next further investigated cell structure by transmission electron microscopy. As shown in Fig. 4D, many more autophagic vesicles were observed in HT-29 cells co-treated with I3C and genistein for 12 h than in untreated cells. As a positive control of autophagy, HT-29 cells were subjected to amino acid starvation for 12 h, showing numerous autophagic vesicles.

### Co-treatment with I3C and genistein does not induce autophagic cell death

**Figure 5:**
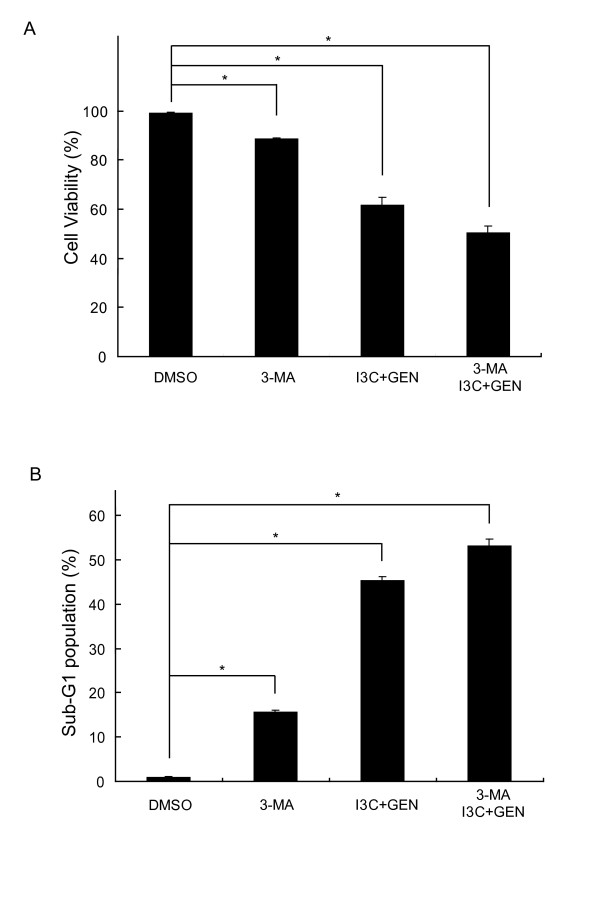
**3-MA does not inhibit the cell death caused by co-treatment with I3C and genistein**. Cell viability (**A**) and the sub-G1 population (**B**) in the HT-29 cells exposed to DMSO, 3-MA (10 mmol/L) or the co-treatment with I3C (300 μmol/L) and genistein (40 μmol/L) with or without 3-MA (10 mmol/L) for 48 h. Cell viability and the sub-G1 population were analyzed as described in Materials and Methods. *Columns*, mean (n = 3); *bars*, SD. *, *P *< 0.05, significantly different compared with the DMSO-treated control.

It has been suggested that excessive autophagy ultimately induces a type of cell death called autophagic cell death (type II programmed cell death) [43]. To investigate whether the cell death caused by the co-treatment with I3C and genistein induces autophagic cell death, cell viability was measured using the autophagy inhibitor 3-MA. As shown in Fig. 5A, 3-MA could not restore cell viability in the cells co-treated with I3C and genistein. We also analyzed sub-G1 population besides the cell viability assay, and found that autophagy inhibition by 3-MA alone or the co-treatment with I3C and genistein clearly induced apoptosis compared to the control, and the apoptosis induced by the co-treatment was not inhibited by 3-MA (Fig. 5B), consistent with the data from Fig. 5A. These results suggest that the cell death caused by the combined treatment with I3C and genistein is not a result of autophagic cell death but apoptosis at least partially through inhibiting autophagic process.

### Co-treatment with I3C and genistein prevents progression of the autophagic process at a later step and disrupts the maturation of autophagosomes

**Figure 6:**
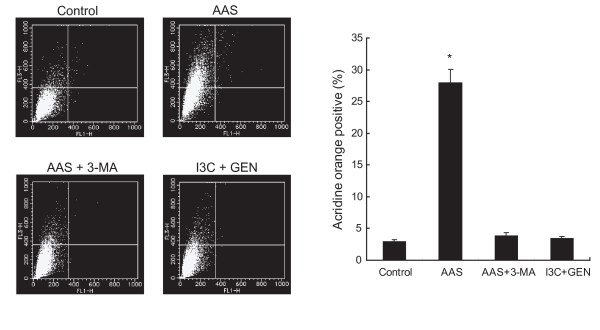
**Co-treatment with I3C and genistein does not induce maturation of autophagosomes**. Quantification of acidic vesicular organelles by acridine orange staining using flow cytometry. HT-29 cells treated with DMSO (control), amino acid-deprived medium (AAS) in the presence or absence of 3-MA (10 mmol/L), or the combination of I3C (300 μmol/L) and genistein (40 μmol/L) for 12 h were stained with acridine orange, and then subjected to a flow cytometric analysis as described in Materials and Methods. *Left*, *FL1-H *indicates the intensity of green fluorescence that is endogenous to the nucleus. *FL3-H *indicates the intensity of red fluorescence. *Top of the grid *was considered as AVOs. *GEN*, genistein. *Right*, *Columns*, mean (n = 3); *bars*, SD. *, *P *< 0.05, significantly different compared with the control.

It has been reported that an enhancement of LC3 expression detected by western blotting does not necessarily reflect an increase in autophagy, but also indicates inhibition of the autophagic process at a later step, resulting in the accumulation of pre-matured autophagosomes [40,44]. Autophagosomes fuse with lysosomes later in the autophagic process forming autolysosomes, the content of which are eventually degraded. The process is known to be accompanied by an increase in the acidity of the lumen followed by the development of acidic vesicular organelles (AVOs) [45]. To quantify the development of AVOs, cells co-treated with I3C and genistein were stained with acridine orange and analyzed by flow cytometry. Acridine orange concentrates in acidic vesicles such as matured autophagosomes including autolysosomes and has been used as an indicator of autophagosomal maturation [27,28,36]. As shown in Fig. 6, in the amino acid-starved cells the strength of the bright red fluorescence (Y axis) increased from 3.0% to 28.0%, indicating the development of AVOs, and 3-MA suppressed the increase from 28.0% to 3.8%, indicating inhibition of the development of AVOs in amino acid-starved cells. The cells co-treated with I3C and genistein did not show significant development of AVOs compared with the control. The results could suggest that co-treatment with I3C and genistein disrupted the maturation of autophagosomes into functional autolysosomes by preventing the progression of the autophagic process at a later stage.

### Apoptosis is enhanced by inhibition of both Akt activity and progression of autophagy

**Figure 7:**
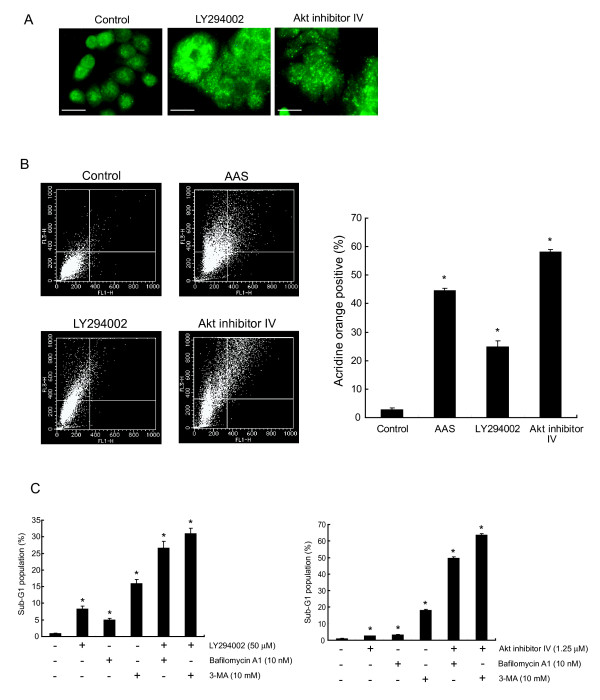
**Effects of simultaneous inhibition of Akt activity and progression of autophagic process in HT-29 cells**. **A**, HT-29 cells were exposed to DMSO (control), LY294002 (50 μmol/L) or Akt inhibitor IV (1.25 μmol/L) for 48 h and then subjected to immunofluorescent staining for LC3 as described in Materials and Methods. *Bars*, 20 μm. **B**, Quantification of acidic vesicular organelles by acridine orange staining using flow cytometry. HT-29 cells treated with DMSO (control), amino acid-deprived medium (AAS), LY294002 (50 μmol/L), or Akt inhibitor IV (1.25 μmol/L) for 48 h were stained with acridine orange and then the development of AVOs was analyzed as described in Materials and Methods. *Left*, *FL1-H *indicates the intensity of green fluorescence that is endogenous to the nucleus. *FL3-H *indicates the intensity of red fluorescence. *Top of the grid *was considered as AVOs. *Right*, *Columns*, mean (n = 3); *bars*, SD. *, *P *< 0.05, significantly different compared with the control. **C**, The sub-G1 population was quantified by flow cytometry after exposure to LY294002 (50 μmol/L) or Akt inhibitor IV (1.25 μmol/L) with or without bafilomycin A1 (10 nmol/L) or 3-MA (10 mmol/L) for 48 h. *Columns*, mean (n = 3); *bars*, SD. *, *P *< 0.05, significantly different compared with the control.

Several studies have suggested that autophagy could protect cells by preventing them from undergoing apoptosis and inhibition of autophagy enhances apoptosis [20,27-36]. From previous reports and the present findings, we hypothesized that the synergistic apoptosis induced by the co-treatment with I3C and genistein might depend on the simultaneous inhibition of Akt activity and the progression of autophagy. To investigate the effects of Akt's inhibition, HT-29 cells were exposed to LY294002 (50 μmol/L), a PI3K-specific inhibitor, or Akt inhibitor IV (1.25 μmol/L), a selective inhibitor of Akt with no effect on PI3K or PDK1. Exposure of LY294002 or Akt inhibitor IV for 48 h changed the localization of LC3 from diffuse cytosolic staining in control cells to a punctate distribution as shown by the immunofluorescent staining of LC3 (Fig. 7A). These results would reflect the development of autophagosomes in the cells treated with LY294002 or Akt inhibitor IV. Moreover, these agents increased the strength of bright red fluorescence compared to the control in the flow cytometric analysis of acridine orange staining, indicating the development of AVOs (Fig. 7B). Collectively, LY294002 and Akt inhibitor IV were thought to enhance the progression of autophagy consistent with previous reports [46,47]. We next measured the sub-G1 population under conditions inhibiting both Akt activity and autophagy. We treated HT-29 cells for 48 h with LY294002 (50 μmol/L) or Akt inhibitor IV (1.25 μmol/L) to inhibit the Akt activity and 3-MA (10 mmol/L) or bafilomycin A1 (10 nmol/L), a specific inhibitor of vacuolar-type H^+^-ATPase, which has been reported to disrupt the progression of autophagy at the later step by inhibiting fusion between autophagosomes and lysosomes [48,49], to inhibit autophagy. As shown in Fig. 7C, inhibition of autophagy by 3-MA or bafilomycin A1 augmented the sub-G1 population in more than an additive fashion in HT-29 cells treated with LY294002 or Akt inhibitor IV. These results give rise to a possibility that inhibition of both Akt activity and autophagy augments apoptosis, consistent with the hypothesis that co-treatment with I3C and genistein synergistically induces apoptosis as a result of the simultaneous inhibition of Akt phosphorylation and autophagy.

## Discussion

Though high doses of single agents have been shown to have potent antitumor effects, the chemopreventive properties of vegetables may result from interactions among several components that potentiate the activities of any single constituent. In the present study, we found a synergistic antitumor effect by co-treatment with I3C and genistein at concentrations more than four times lower than those of each agent alone (Fig. 1A and 1B). We concluded that the antitumor effect was due to apoptosis via inhibition of both Akt phosphorylation and the progression of autophagy.

The PI3K/Akt pathway has been reported to play an important role in the inhibition of apoptosis [13,14]. Once activated, Akt phosphorylates and inactivates several proapoptotic proteins, including Bad [15] and caspase-9 [16], thus inhibiting intrinsic apoptotic pathway. Akt also inhibits the extrinsic death receptor-mediated apoptotic pathway through up-regulation of FLICE inhibitory protein (FLIP) expression [50,51], which can inhibit apoptosis as an antagonist of caspase-8 [52]. Akt thus inhibits apoptosis by suppressing both the intrinsic and extrinsic pathways. Moreover, recent studies have suggested that XIAP or survivin is positively regulated by Akt [17,18]. It has been also reported that I3C or genistein alone inhibits the phosphorylation of Akt [4,10].

However, in the present study, neither agent alone reduced the phosphorylation of Akt, whereas co-treatment with I3C and genistein did (Fig. 3). We have also found that caspase-9, known as a downstream target of Akt, was dephosphorylated and cleaved to the active form by the combined treatment, as expected. In addition, we found the cleavage of caspase-8 by the combination treatment. The combination also caused a reduction in XIAP and survivin. Collectively, these results suggest that the activation of caspase-9 and caspase-8 with suppression of XIAP and survivin expressions via inhibition of the Akt pathway contribute, at least in part, to the apoptotic cell death caused by the co-treatment.

Genistein is known as one of the major phytoestrogens that are structurally similar to estradiol, binding to estrogen receptor β (ERβ) with considerably higher affinity than to estrogen receptor α (ERα)[53]. However, it is still unknown whether the antiproliferative effects of genistein in colon cancer cells involve the transcriptional regulation mediated by estrogen receptors in addition to the tyrosine kinase pathway [9]. I3C and its metabolite diindolylmethane (DIM) are known androgen receptor antagonist and DIM is also an ER agonist like genistein [12]. Both I3C and DIM caused anti-proliferative effects on prostate cancer cells via AR-mediated pathway [12]. In addition, both ER and AR are expressed in normal intestine, including the colon [54]. However, in HT-29 cells, the expression of ERα, ERβ, and AR protein levels was undetectable [55]. We therefore consider that the anti-proliferative effect by the combination of I3C with genistein is independent of the nuclear receptor pathways.

mTOR is another downstream target of Akt, and inhibition of the PI3K/Akt/mTOR pathway has been shown to initiate autophagy [19,37-39]. Increasing evidence has suggested that several flavonoids induce autophagy [56,57]. We next found that co-treatment with I3C and genistein also caused dephosphorylation of mTOR, associated with the formation of autophagosomes (Fig. 4C and 4D). At the same time, we found that the progression of the autophagic process was inhibited by the combination as mentioned below.

Several studies have suggested that inhibition of the maturation of autophagosomes causes the accumulation of pre-matured autophagosomes [29,30,40,44]. The maturation of autophagosomes into autolysosomes is accompanied by an increase in AVOs reflecting the acidity of the lumen [45]. We found that the combination of I3C and genistein did not develop AVOs, suggesting the maturation of autophagosomes to be inhibited (Fig. 6). Additionally, we found the accumulation of LC3-II (Fig. 4A and 4B) consistent with a report that inhibition of the autophagic process at the maturation step enhances LC3-II expression [40]. Therefore, co-treatment with I3C and genistein might promote the formation of autophagosome, but prevent their maturation, though the precise mechanism remains to be elucidated.

The role of the autophagic process in antitumor therapy has not been clearly elucidated. To adapt to the stressful conditions caused by anticancer therapies, cancer cells undergo autophagy as a temporary survival mechanism. The suppression of autophagy leads to apoptosis, thus enhancing the antitumor effect [29-35]. On the other hand, several anticancer treatments, including irradiation and chemotherapeutic agents, have been reported to induce autophagic cell death [20,27,38], which has no characteristics of apoptosis, indicating autophagy to be a crucial mechanism of the cancer cell death caused by these treatments. In the present study, an autophagy inhibitor 3-MA could not reverse the loss of cell viability among HT-29 cells caused by the combined treatment, suggesting that the treatment does not induce autophagic cell death (Fig. 5). The results raise the possibility that the suppression of autophagy by the co-treatment causes apoptosis.

In the present study, we found that inhibition of both Akt phosphorylation and the progression of autophagy enhanced apoptosis in HT-29 cells (Fig. 7C). Degtyarev *et al*. have also demonstrated that blocking autophagy with bafilomycin A1 enhanced apoptosis in tumor cells when Akt activity was inhibited [58]. This report supports our hypothesis that the mechanism underlying the synergistic induction of apoptosis by the co-treatment with I3C and genistein results from simultaneous inhibition of the PI3K/Akt pathway and progression of the autophagic process.

Sato *et al*. have suggested that autophagy is activated in colorectal cancer *in vitro *and *in vivo*, and might contribute to the survival of cancer cells [59]. Additionally, they found a remarkable enhancement of apoptosis by inhibiting autophagy. Bauvy *et al*. have reported that autophagy delays sulindac sulfide-induced apoptosis in HT-29 cells, suggesting autophagy to be a protective mechanism in this cell line [34]. These reports suggest that stimulation of autophagy may lead to enhanced tumor growth and that therapies inhibiting autophagy may be effective against colon cancer cells.

Finally, it is important to discuss physiological concentrations and toxicity of I3C and genistein. According to the previous reports, the plasma level of DIM converted from I3C in people who received oral doses of 1 g I3C was almost 1-5 μmol/L [60]. They have also shown that I3C itself was not detected in plasma and the only DIM was detected. Firestone *et al*. reported that I3C is converted intracellularly into DIM in the cultured breast cancer cells [61]. Furthermore, Bonnesen *et al*. indicated that the DIM was about 9-15 times more toxic to human colon cancer cells than I3C [62]. However, it is unknown how much I3C is converted into DIM in the cultured HT-29 cells, and we can hardly evaluate the physiological effects of I3C. When 2, 4, or 8 mg/kg of genistein was orally administered to human, the plasma concentrations of genistein were 4.3-16.3 μmol/L [63]. Busby MG *et al*. reported that the maximum plasma concentrations of total genistein were 27.46 ± 15.38 μmol/L when 16 mg/kg of genistein was orally administered [64]. In this regard, 40 μmol/L of a plasma genistein may be achievable with oral intake of purified genistein. They also reported toxic effects including hypophosphatemia, pedal edema and so on, though they were not associated with clinical toxicity. Since their observation is limited to a short period, further investigation of the toxicology over longer periods must be conducted before clinical usage.

Although genistein and I3C are known to have antitumor effects extensively, they also have been reported to stimulate carcinogenesis or growth of tumors. Genistein has been found to inhibit the growth of human breast cancer MCF-7 cells at high concentrations (above 20 μmol/L) [65], but to stimulate the growth at a lower concentration (200 nmol/L) [66]. Regarding *in vivo *studies, genistein has been shown to have preventive effect in the azoxymethane-induced rat colon carcinogenesis model [67], whereas genistein increased aberrant crypt foci by 1,2-dimethylhydrazine in rats fed diets containing genistein [68]. On the other hand, I3C is known to exhibit chemopreventive effects in experimental animal models, such as a spontaneous occurrence of endometrial carcinoma in female Donryu rats [69], 7,12-dimethylbenz (a) anthracene (DMBA) or *N*-methyl-*N*-nitrosourea (MNU) induced rat carcinogenesis [70], or an aflatoxin B1-induced rat carcinogenesis [71]. However, several reports have suggested that I3C enhances carcinogenesis in certain protocols, such as a rat multiorgan carcinogenesis by sequential treatment with diethylnitrosoamine (DEN), MNU and dihydroxy-di-N-propyl-nitrosoamine (DHPN) [72], or a rat 1,2-dimethylhydrazine (DMH) induced colon carcinogenesis [73]. Collectively, effects of genistein and I3C on malignant tumors are not fully established. Therefore, the efficacy and risk potential of these dietry components must be considered with careful attention, and further investigation is required.

## Conclusion

The present study is the first to show the efficacy of combined treatment with naturally occurring flavonoids which inhibit the PI3K/Akt pathway and autophagic process. Although further studies are required to examine the adverse health effects of the combination treatment with I3C and genistein, including stimulating the induction and growth of tumors, we believe that the present study could be a clue to a novel strategy against malignant tumors using dietary phytochemicals.

## Methods

### Reagents

Genistein was purchased from Fujicco (Kobe, Japan). Indole-3-carbinol, 3-methyladenine (3-MA), bafilomycin A1, ribonuclease A (RNase A), propidium iodide, anti-β-actin antibody and anti-LC3B antibody were purchased from Sigma (Saint Louis, MO). LY294002, anti-phospho-Akt (Ser473), anti-Akt, anti-phospho-mTOR (Ser2448), anti-mTOR, anti-poly (ADP-ribose) polymerase (PARP), and anti-cleaved caspase-3 antibodies were purchased from Cell Signaling Technology (Beverly, MA). Z-VAD-fmk, anti-XIAP, anti-survivin, anti-caspase-9 and anti-caspase-8 antibodies were purchased from R&D Systems (Minneapolis, MN). Akt inhibitor IV was purchased from Carbiochem (San Diego, CA). Anti-phospho-caspase-9 antibody was obtained from Santa Cruz Biotechnology (Santa Cruz, CA).

### Cell culture

Human colon cancer HT-29 cells were cultured in Dulbecco's modified Eagle's medium (DMEM) as described previously [11]. For amino acid starvation, HT-29 cells were maintained in amino acid-deprived DMEM purchased from Cell Science & Technology Institute, Inc. (Sendai, Japan).

### Cell viability assay

HT-29 cells were seeded at 1.2 × 10^4 ^per well in 24-well culture plates and incubated for 24 h. The cells were then exposed to the indicated agents for the indicated times, and cell viability was analyzed using a Guava EasyCyte plus flow cytometer according to the manufacturer's instructions (Guava Technologies Inc.).

### Drug interaction analysis

The effect of drug combination was evaluated by combination index (CI) method using the CalcuSyn software (Biosoft, Ferguson, MO), which is based on the median effect model of Chou and Talalay [74]. HT-29 cells were exposed to I3C at concentrations ranging from 200 μmol/L to 600 μmol/L and to genistein at 26.7 μmol/L to 80 μmol/L, either alone or in a fixed ratio of 7.5:1 (I3C:genistein), for 48 h. Then the cell viability assay was performed as described above and data obtained from the assay were used to calculate values of fraction affected (Fa) using the following formula: Fa = 1 - (cell viability of treated group (%)/cell viability of control group (%)). Such experimental data were entered into the CalcuSyn interface and used to calculate combination index (CI) values. Serial CI values over an entire range of drug-effect levels (Fa) were then calculated. These data were used to generate Fa-CI plots, from which synergy or antagonism can be identified. Synergy, additivity, and antagonism are defined as CI<1, CI = 1, and CI>1, respectively.

### Detection of apoptosis

For the detection and analysis of apoptosis, the nuclei of cells were stained with propidium iodide and measured using Becton Dickinson FACSCalibur as described previously [11]. For the observation of nuclear morphology, cells treated under various conditions as indicated were fixed in methanol, incubated with 4',6-diamidino-2-phenylindole (DAPI) solution, and then analyzed using a fluorescence microscope (IX-70; Olympus, Tokyo, Japan).

### Western blotting

Cells were lysed in RIPA buffer containing PhosSTOP (Roche Applied Science, Mannheim, Germany). Fifty micrograms of protein was resolved by 7.5%, 10% or 15% SDS-PAGE and transferred to PVDF membranes (Millipore, Bedford, MA). After being blocked, the membranes were incubated with primary antibody. After washing, the membranes were incubated with HRP-conjugated secondary antibody, as described previously [11]. The signals were detected with the ECL western blot analysis system (GE Healthcare, Piscataway, NJ).

### Transmission electron microscopy

The treated cells were collected by trypsinization and fixed with 1.5% glutaraldehyde for 2 h at 4°C and postfixed with 2% osmium tetroxide for 2 h. After dehydration with 50% to 100% alcohol, the cells were embedded in Quetol 812 resin (Nissin EM, Tokyo, Japan). After polymerization, ultrathin sections (80 nm) were collected on a copper grid and stained with uranyl acetate for 15 min, followed by lead citrate for 5 min, then representative areas were observed under an electron microscope (H-300, Hitachi, Tokyo, Japan).

### Immunofluorescent staining of LC3

After treatment under various conditions as indicated, the cells were fixed with 4% paraformaldehyde for 10 min, permeabilized with 100 μg/ml of digitonin, rinsed three times with PBS, blocked by 1% BSA for 1 h, and incubated with an anti-LC3 antibody (MBL, Nagoya, Japan) for 1 h at room temperature. The cells were washed, incubated with FITC-conjugated secondary antibody for 30 min at room temperature, washed again and observed under a fluorescence microscope (IX-70, Olympus).

### Quantification of acidic vesicular organelles (AVO) with acridine orange staining

To quantify the development of AVOs, we performed vital staining with acridine orange as described previously [27,28,36]. Briefly, HT-29 cells were stained with acridine orange (1 μg/mL) for 15 min, collected by trypsinization, washed with PBS and analyzed with FACSCalibur.

### Statistical analysis

Data were expressed as means ± SD for triplicate experiments. The statistical evaluation of the data was done using Student's *t *test for simple comparison between groups and treatments. *P *< 0.05 was considered statistically significant.

## Abbreviations

AVOs: acidic vesicular organelles; AR: androgen receptor; CI: combination index; DIM: diindolylmethane; DMSO: dimethyl sulfoxide; ERα or β: estrogen receptor α or β; GEN: genistein; I3C: indol-3-carbinol; LC3: microtubule-associated protein-1 light chain-3; mTOR: mammalian target of rapamycin; PARP: poly (ADP-ribose) polymerase; XIAP: X chromosome-linked inhibitor of apoptosis protein; 3-MA: 3-methyladenine.

## Competing interests

The authors declare that they have no competing interests.

## Authors' contributions

YN conceived of the studies, carried out all experiments described in this paper, performed statistical studies and drafted the manuscript. SY, YI and HW participated in western blot analysis. SY, EO and TS coordinated this study and helped draft and edit the manuscript. All authors read and approved the final manuscript.

## Supplementary Material

Additional file 1 **Time-dependent expression of cleaved caspase-3**. After exposure to DMSO (control), I3C (300 μmol/L), genistein (40 μmol/L) or a combination of I3C (300 μmol/L) and genistein (40 μmol/L) for the periods indicated, cell lysates were subjected to western blotting with an anti-cleaved caspase-3 antibody. β-actin was used as a loading control. -, treated with DMSO. *GEN*, genistein. Click here for file
