# Supplementary figures and images for: A combination of indol-3-carbinol and genistein synergistically induces apoptosis in human colon cancer HT-29 cells by inhibiting Akt phosphorylation and progression of autophagy
Source: Mol Cancer. 2009 Nov 12;8:100. doi: 10.1186/1476-4598-8-100 (PMC2784428; doi:10.1186/1476-4598-8-100)

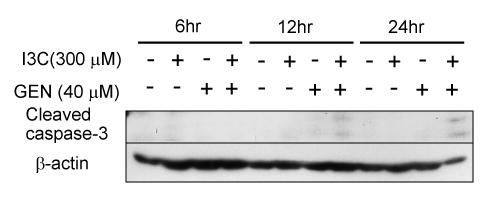

Supplement: Additional file 1 — Time-dependent expression of cleaved caspase-3. After exposure to DMSO (control), I3C (300 μmol/L), genistein (40 μmol/L) or a combination of I3C (300 μmol/L) and genistein (40 μmol/L) for the periods indicated, cell lysates were subjected to western blotting with an anti-cleaved caspase-3 antibody. β-actin was used as a loading control. -, treated with DMSO. GEN, genistein. [file 1476-4598-8-100-S1.doc]
